# Multi-omics analysis of TLCD1 as a promising biomarker in pan-cancer

**DOI:** 10.3389/fcell.2023.1305906

**Published:** 2024-03-15

**Authors:** Shengli Wang, Mingyue Zhang, Hongyan Sun, Tao Li, Jianlei Hao, Meixia Fang, Jie Dong, Hongbiao Xu

**Affiliations:** ^1^ Zhuhai Institute of Translational Medicine, Zhuhai People’s Hospital Affiliated With Jinan University, Jinan University, Zhuhai, Guangdong, China; ^2^ The Biomedical Translational Research Institute, Faculty of Medical Science, Jinan University, Guangzhou, Guangdong, China; ^3^ Department of Laboratory Animal, Institute of Laboratory Animal Science, Jinan University, Guangzhou, Guangdong, China; ^4^ Department of Clinical Laboratory, Guangzhou Twelfth People’s Hospital, Guangzhou, Guangdong, China; ^5^ Department of Thyroid and Breast Surgery, The First Affiliated Hospital, Sun Yat-sen University, Guangzhou, Guangdong, China

**Keywords:** TLCD1, bioinformatics, prognosis, immune infiltration, immunotherapy response

## Abstract

**Background:** The TLC Domain Containing 1 (TLCD1) protein, a key regulator of phosphatidylethanolamine (PE) composition, is distributed across several cellular membranes, including mitochondrial plasma membranes. Existing research has revealed the impact of TLCD1 on the development of non-alcoholic fatty liver disease. However, there remains a gap in comprehensive pan-cancer analyses of TLCD1, and the precise role of TLCD1 in cancer patient prognosis and immunological responses remains elusive. This study aims to provide a comprehensive visualization of the prognostic landscape associated with TLCD1 across a spectrum of cancers, while shedding light on the potential links between TLCD1 expression within the tumor microenvironment and immune infiltration characteristics.

**Methods:** TLCD1 expression data were obtained from GTEx, TCGA, and HPA data repositories. Multiple databases including TIMER, HPA, TISIDB, cBioPortal, GEPIA2, STRING, KEGG, GO, and CancerSEA were used to investigate the expression pattern, diagnostic and prognostic significance, mutation status, functional analysis, and functional status of TLCD1. In addition, we evaluated the relationship between TLCD1 expression and immune infiltration, tumor mutational burden (TMB), microsatellite instability (MSI), and immune-related genes in pan-cancer. Furthermore, the association of TLCD1 with drug sensitivity was analyzed using the CellMiner database.

**Results:** We found that TLCD1 is generally highly expressed in pan-cancers and is significantly associated with the staging and prognosis of various cancers. Furthermore, our results also showed that TLCD1 was significantly associated with immune cell infiltration and immune regulatory factor expression. Using CellMiner database analysis, we then found a strong correlation between TLCD1 expression and sensitivity to anticancer drugs, indicating its potential as a therapeutic target. The most exciting finding is that high TLCD1 expression is associated with worse survival and prognosis in GBM and SKCM patients receiving anti-PD1 therapy. These findings highlight the potential of TLCD1 as a predictive biomarker for response to immunotherapy.

**Conclusion:** TLCD1 plays a role in the regulation of immune infiltration and affects the prognosis of patients with various cancers. It serves as both a prognostic and immunologic biomarker in human cancer.

## 1 Introduction

Cancer is a major global health challenge and a leading cause of morbidity and mortality worldwide (Iskandar et al., 2021). Cancer research has made significant progress in unravelling key oncogenic pathways that are intricately linked to critical cellular processes, including signaling, apoptosis, cell cycle checkpoints and histone modifications (Singh and McGuirk, 2020), and various treatment modalities, ranging from radiotherapy and chemotherapy to targeted therapy and immunotherapy, have emerged as critical tools in our fight against cancer. These ongoing research efforts have not only deepened our understanding of the intricate pathogenesis of tumors, but have also led to significant advances in treatment strategies. However, it is important to note that immunotherapy, while promising, requires further comprehensive investigation across multiple cancer types to fully substantiate its efficacy (Yang, 2015; Srivastava and Hanash, 2020). Pan-cancer analysis, which involves the study of genes across multiple cancer types, is a valuable approach to identify both commonalities and differences in gene expression patterns (Srivastava and Hanash, 2020). To facilitate these analyses, public databases such as The Cancer Genome Atlas (TCGA) provide a wealth of data that can be used for purposes such as cancer diagnosis, prognosis and the development of immunotherapeutic interventions (Blum et al., 2018).

Some researchers have observed in HeLa cells that TLCD1 is localized in the vicinity of mitochondria, most likely on mitochondria-associated membranes (Petkevicius et al., 2022). The TLCD1 protein regulates long-chain polyunsaturated fatty acid (LCPUFA) phospholipid levels in the plasma membrane (Ruiz et al., 2018). In human physiology, LCPUFAs are substances with a wide range of central structural and regulatory functions and are actively involved in a variety of biological processes (Bannenberg et al., 2017), and occupy a crucial position as key components in complex lipid molecules (Castro et al., 2016; Zhu et al., 2019). TLCD1 is a key regulator of phosphatidylethanolamine (PE) composition. However, lipids play a key role in various stages of tumorigenesis and disease progression, including the destruction of normal tissue structure, cancer cell migration, and the interaction between cancer cells and tumor microenvironment components interactions and reprogramming of lipid metabolism in cancer cells (Joyce and Pollard, 2009; Park et al., 2012; Beloribi-Djefaflia et al., 2016). Lipid alterations have been strongly implicated in the initiation and progression of a variety of cancers, including lung cancer (Smith et al., 2008), breast cancer (Dória et al., 2012) and colon cancers (Fhaner et al., 2012). The removal of TLCD1 leads to an increased uptake of unsaturated fatty acids, ultimately contributing to the restoration of cell membranes to a healthy state, even in the presence of excess saturated fats in the culture medium (Ruiz et al., 2018). These findings open up new perspectives and opportunities in the field of cancer research and therapy, with profound implications for the understanding and treatment of various diseases.

In this study, we used a variety of bioinformatics techniques to perform a comprehensive pan-cancer analysis of TLCD1. Our analysis included multiple dimensions, including gene expression, genomic alterations, associations with prognosis, immune markers, immune infiltration and relevant gene sets. Our results show a significant correlation between TLCD1 expression and immune responses, suggesting its potential as a valuable prognostic biomarker across multiple cancer types.

## 2 Materials and methods

### 2.1 TLCD1 expression analysis

The mRNA and protein expression levels of TLCD1 in various normal human tissues were analyzed using the GTEx Portal database and The Human Protein Atlas database. The TLCD1 gene expression in various cancer tissues was investigated by accessing the “Gene_DE” module of the Tumor ImmunoEstimation Resource 2.0 (TIMER2) website (https://timer.cistrome.org/) and utilizing the Gene Expression Profiling Interactive Analysis (GEPIA) databases. RNA-seq data for both normal and tumor samples were obtained from the TCGA and GTEx projects.

### 2.2 Analysis of tumor stage and survival prognosis

We used R software (version 3.6.4) to calculate the expression differences of genes in different clinical stage samples within each tumor. Non-paired Wilcoxon Rank Sum and Signed Rank Tests were employed for pairwise significance analysis between samples, and the Kruskal–Wallis Test was used for differential testing among multiple sample groups. In addition, survival plots showing overall survival (OS) and disease-free survival (DFS) for TLCD1 in different tumor types were obtained from the TCGA database via the GEPIA2 online platform. Expression thresholds of 50% were applied to categorize high and low TLCD1 expression groups, resulting in the formation of different cohorts based on TLCD1 expression levels.

### 2.3 Analysis of genetic alterations

Performed a detailed analysis of genetic alterations in TLCD1 in various cancers using the cBioPortal tool. The TCGA Pan-Cancer Atlas Study was selected as the primary cohort for this analysis. The mutational landscape of TLCD1, including the type and frequency of mutations, was comprehensively assessed using the OncoPrint and CancerTypes Summary features of cBioPortal. The OncoPrint feature visually displays mutations, copy number variations and gene expression levels of TLCD1 across all samples in the form of heat maps. A comprehensive investigation into the proportion of TLCD1 gene mutations in various tumor types was conducted using the Gene Mutation module of TIMER 2.0. Specifically, “TLCD1” was entered into the “Query” module to access details on altered loci, mutation types, and the total number of TLCD1 mutations across different cancer types. Furthermore, the standardized pan-cancer dataset from the UCSC database, namely, the TCGA TARGET GTEx dataset (PANCAN, N = 19131, G = 60499), was obtained. Expression data for 44 marker genes associated with TLCD1 and three RNA modification types (m1A, m5C, m6A) were extracted for each sample, followed by a log2(x + 0.001) transformation of each expression value. Pearson correlations between TLCD1 and five markers of the immune pathway were calculated. Integration of TCGA-level simple nucleotide variant datasets for the samples was performed using MuTect2 software downloaded from the GDC. Additionally, the tumor mutation burden (TMB) was calculated for each tumor using the TMB function in the R package map tools. The integration of TMB and gene expression data for the samples provided a comprehensive overview of the genetic landscape. This multifaceted analysis enabled valuable insights into genetic alterations and immune-related effects associated with TLCD1 across different cancer types.

### 2.4 Analysis of immune infiltration

The detailed analysis of immune infiltration involved the extraction of TLCD1 gene expression data from each sample within the pan-cancer dataset obtained from the UCSC database. The tumor microenvironment assessment was conducted using the R package ESTIMATE, enabling the calculation of stromal, immune, and total ESTIMATE scores for each patient within each tumor based on their gene expression profiles. These scores offer valuable insights into the degree of infiltration by stromal and immune cells in the tumor microenvironment. Ultimately, immune infiltration scores were obtained for a total of 10,180 tumor samples across 44 different tumor types. Pearson correlation analyses were performed to identify genes exhibiting significant correlations with immune infiltration scores within each tumor. The objective of these analyses was to reveal meaningful associations between genes and immune infiltration scores, providing insight into the intricate relationship between tumor genetics and the tumor microenvironment across various cancer types.

### 2.5 Immune checkpoints and tumor stemness score analysis

An analysis was conducted using the TLCD1 gene and 60 marker genes from two different categories of immune checkpoint pathways, namely, inhibitory (Ozga et al., 2021) and stimulatory (Thorsson et al., 2018). These marker genes were extracted from the pan-cancer dataset obtained from the UCSC database. Pearson correlations were calculated for each sample to explore potential associations, utilizing TLCD1 genes from the same pan-cancer dataset obtained from the UCSC database. DNAs data calculated from methylation profiles for each tumor were combined with these correlations to compute the tumor stemness score. Incorporating the stemness index and gene expression data for the samples, a further step involved performing a log2(x + 0.001) transformation for each expression value to enhance the analysis. Cancer types with fewer than three samples within a given category were filtered out, resulting in the extraction of expression data for 37 different cancer types. The objective of these analyses was to uncover potential relationships between TLCD1, immune checkpoint genes, and tumor stemness scores, aiming to provide valuable insights into the complex interplay between these factors within various cancer types.

### 2.6 Function and enrichment analysis

The functional aspects of TLCD1 were investigated by conducting gene co-expression analysis using the LinkedOmics platform. For this analysis, the HiSeq RNA platform and the TCGA_CESC cohort were selected. Pearson’s test was employed to detect correlations between TLCD1 and its co-expressed genes, and the relationship between TLCD1 and the functional status of 14 different cancers was further investigated. These analyses provide valuable insights into the functional associations of TLCD1 in cancer contexts and allow us to uncover potential roles and implications of TLCD1 in different cancer types.

### 2.7 Analysis of TLCD1-related genes

Experimentally determined TLCD1-binding proteins (*Homo sapiens*) were retrieved by conducting a comprehensive analysis of genes associated with TLCD1 using the STRING database. Interaction parameters included a minimum required low confidence interaction score (0.150), network type as a complete network, and a maximum of 50 interactors. Network edges were determined based on available evidence. The top 100 TLCD1-related target genes were identified using the “similar gene detection” module within GEPIA2. The correlation between TLCD1 and the top 10 genes was analyzed using the “Correlation Analysis” module of GEPIA2. Pearson correlation coefficients were calculated and the results were presented in dot plots. The expression levels of the top 10 target genes in each tumor were obtained using the Gene_Corr module of TIMER2.0. These expression levels were displayed as a heat map. For gene set enrichment analysis, the latest KEGG Pathway gene annotations were accessed through the KEGG Rest API, and the genes were mapped to the background collection. Enrichment analysis was performed using the R software package clusterProfiler (version 3.14.3). Gene sets meeting the criteria of a minimum of 5 genes, a maximum of 5,000 genes, a *p* value < 0.05, and an FDR <0.05 or <0.25 were considered to be statistically significant. The “c5” gene set from the Molecular Signature Database (MSigDB) was downloaded and utilized as the background for the enrichment analysis. Subsequently, the genes were mapped to this background, and the enrichment analysis was conducted using the R software package clusterProfiler (version 3.14.3). Significant results were determined with a minimum gene set of 5, a maximum gene set of 5,000, and a *p* value < 0.05 and FDR <0.05 or <0.25. This comprehensive analysis allowed us to explore TLCD1-related genes, their interactions and potential functional implications, providing valuable insights into the role of TLCD1 in various biological processes and pathways.

### 2.8 Drug sensitivity of TLCD1 in pan-cancer

To analyze the drug sensitivity of TLCD1 in pan-cancer, NCI-60 compound activity data and RNA-seq expression profiles were downloaded into CellMiner™ (Rizvi et al., 2015). FDA-approved or clinical trial drugs were selected for analysis using R version 4.1.3.

### 2.9 Statistical analysis

The Wilcoxon test was utilized to assess differential expression between tumors and adjacent normal tissues in TIMER. Additionally, the Mann-Whitney U test was employed to analyze the immunohistochemistry (IHC) results obtained from the HPA database. For the comparison of survival curves, the log-rank test was utilized to calculate the Hazard Ratio (HR) and log-rank *p*-value in the Kaplan-Meier Plotter. Moreover, Spearman’s correlation analysis was conducted to assess the correlation of gene expression. Significance was marked as follows: *p < 0.05, **p < 0.01, ***p < 0.001, and ****p < 0.0001.

## 3 Results

### 3.1 Tissue-specific expression of TLCD1

**FIGURE 1:**
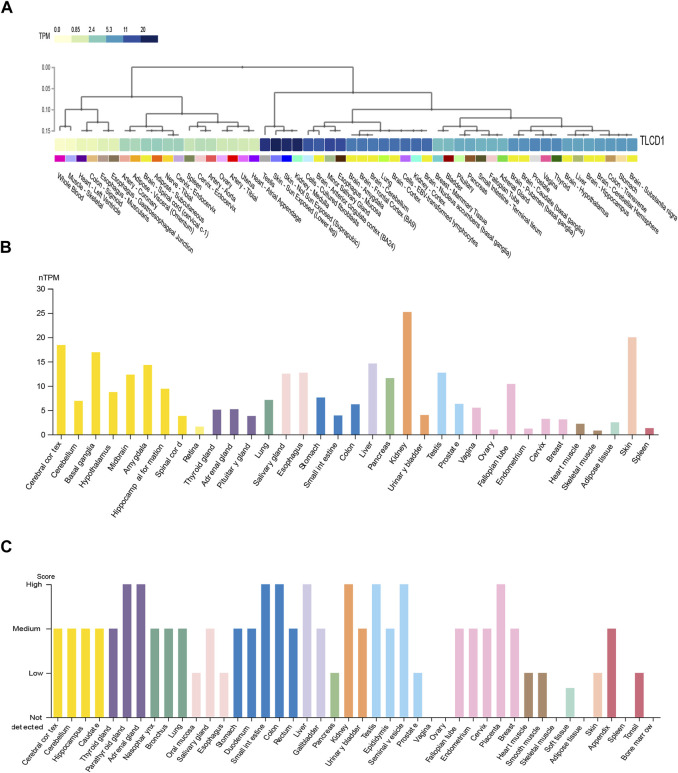
The expression levels of TLCD1 in various human normal tissues. **(A,B)** TLCD1 mRNA expression data generated from GTEx and HPA databases, respectively. **(C)** TLCD1 protein expression profiles in human normal tissues.

In our initial investigation, we evaluated both mRNA and protein expression patterns of TLCD1 in various human tissues using data from the GTEx portal and HPA databases. The results revealed distinct expression profiles in different tissues. Specifically, we observed that TLCD1 mRNA expression was particularly high in tissues such as kidney, skin, testis and cerebral cortex (Figures 1A, B). At the protein level, increased TLCD1 expression was observed in tissues such as thyroid, small intestine, colon, kidney, seminal vesicle, and placenta (Figure 1C). In conclusion, our analysis underscores the tissue-specific nature of TLCD1 expression, with varying levels observed in different tissue types.

### 3.2 Aberrant expression of TLCD1 in multiple tumors

**FIGURE 2:**
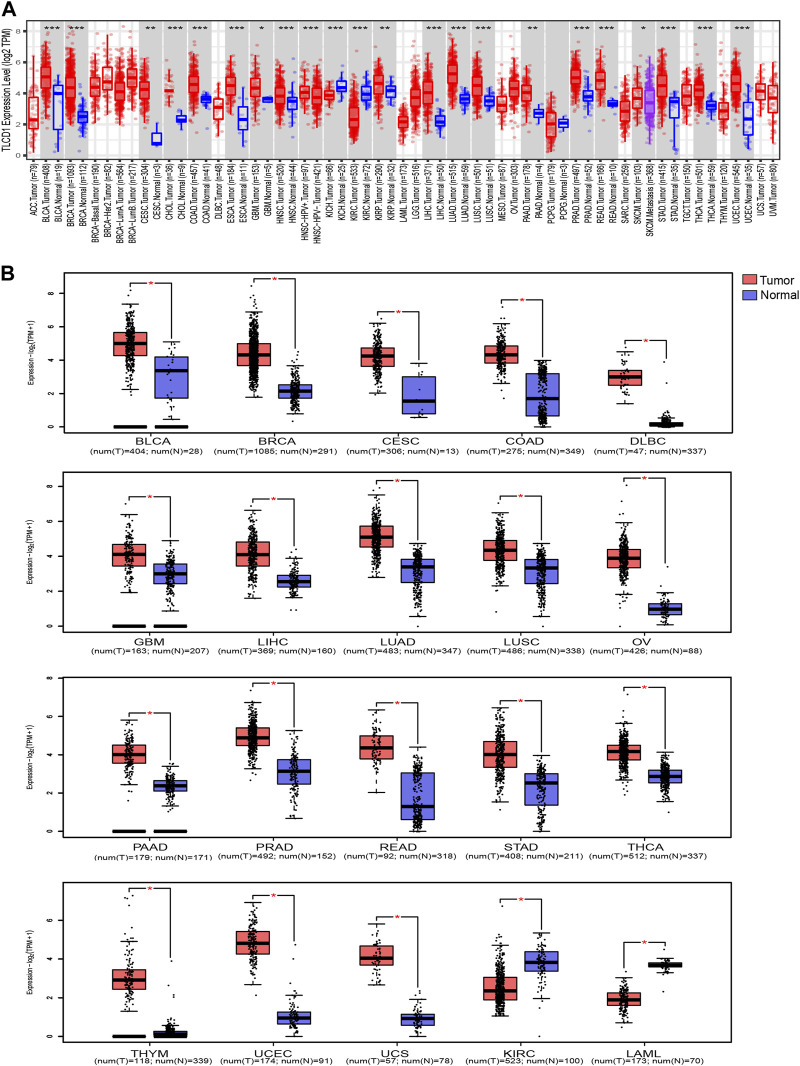
Differential expression of TLCD1 in various tumors. **(A)** TLCD1 expression levels in pan-cancer from TCGA database were analyzed by TIMER2.0. (*p < 0.05, **p < 0.01, ***p < 0.001). **(B)** Box plots of TLCD1 expression levels in different tumors of BLCA, BRCA, CESC, COAD, DLBC, GBM, LIHC, LUAD, LUSC, OV, PAAD, PRAD, READ, STAD, THCA, THYM, UCEC, UCS, KIRC, and LAML (TCGA and GTEx databases). *p < 0.05.

**FIGURE 3:**
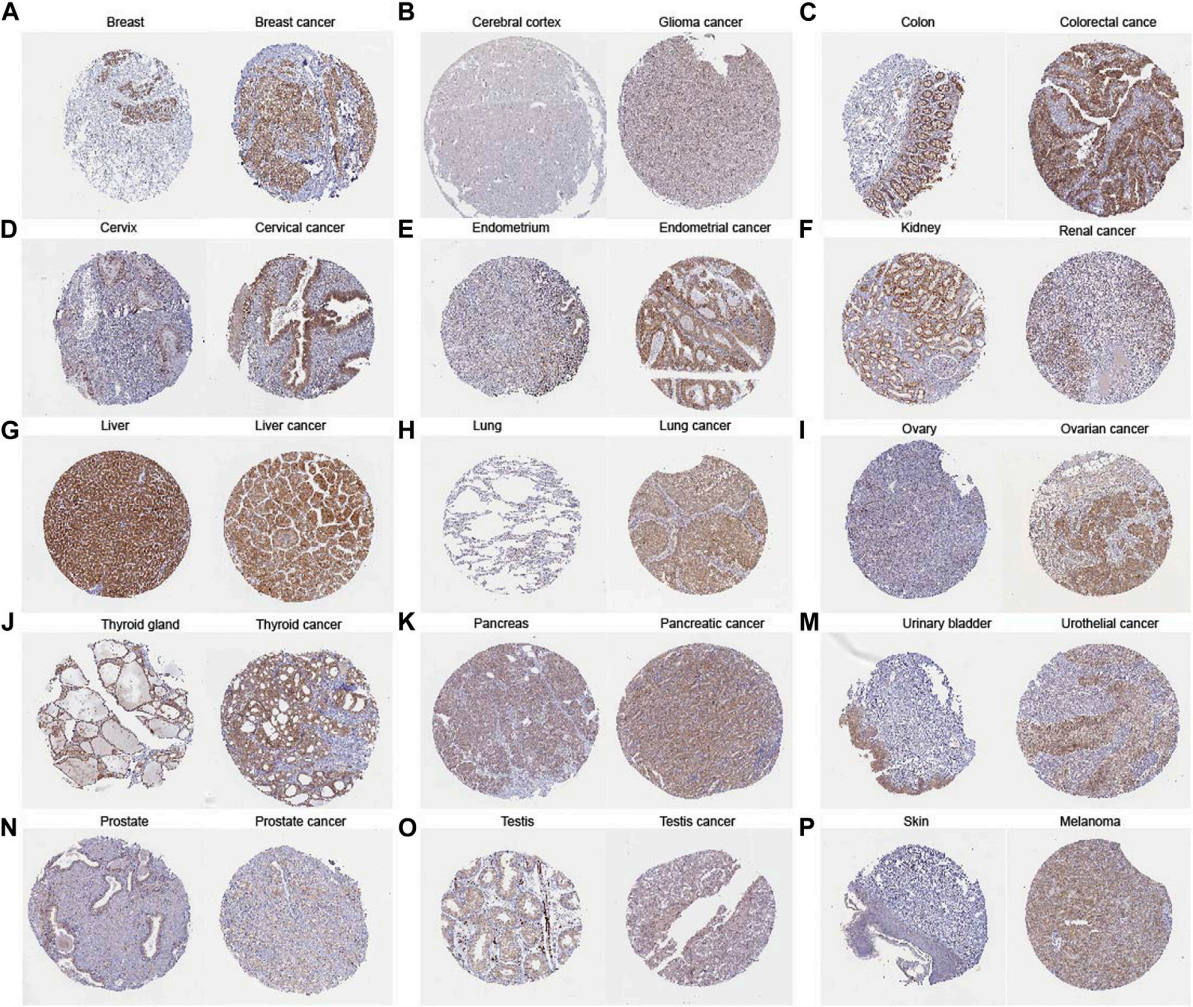
Comparison of TLCD1 gene expression between normal (left) and tumor (right) tissues of immunohistochemistry images **(A–P)**.

Our comprehensive analysis, integrating data from both the TCGA and GTEx databases, revealed distinct and tumor-specific expression patterns of TLCD1 in 33 different cancer tissues. TLCD1 was significantly upregulated in a number of tumors, including BLCA (Bladder Urothelial Carcinoma), BRCA (Breast invasive carcinoma), CESC (Cervical squamous cell carcinoma and endocervical adenocarcinoma), COAD (Colon adenocarcinoma), DLBC (Lymphoid Neoplasm Diffuse Large B cell Lymphoma), GBM (Glioblastoma multiforme), LIHC (Liver hepatocellular carcinoma), LUAD (Lung adenocarcinoma), LUSC (Lung squamous cell carcinoma), OV (Ovarian serous cystadenocarcinoma), PAAD (Pancreatic adenocarcinoma), PRAD (Prostate adenocarcinoma), READ (Rectum adenocarcinoma), STAD (Stomach adenocarcinoma), THCA (Thyroid carcinoma), THYM (Thymoma), UCEC (Uterine Corpus Endometrial Carcinoma), and UCS (Uterine Carcinosarcoma), compared to their respective normal tissues (Figures 2A, B). Notably, TLCD1 expression was found to be reduced in KIRC(Kidney renal clear cell carcinoma) and LAML (Acute Myeloid Leukemia) tumors. Immunohistochemistry showed that TLCD1 expression was higher in most tumor tissues than in normal tissues (Figure 3).

### 3.3 Correlation of TLCD1 expression with tumor stage and prognosis

**FIGURE 4:**
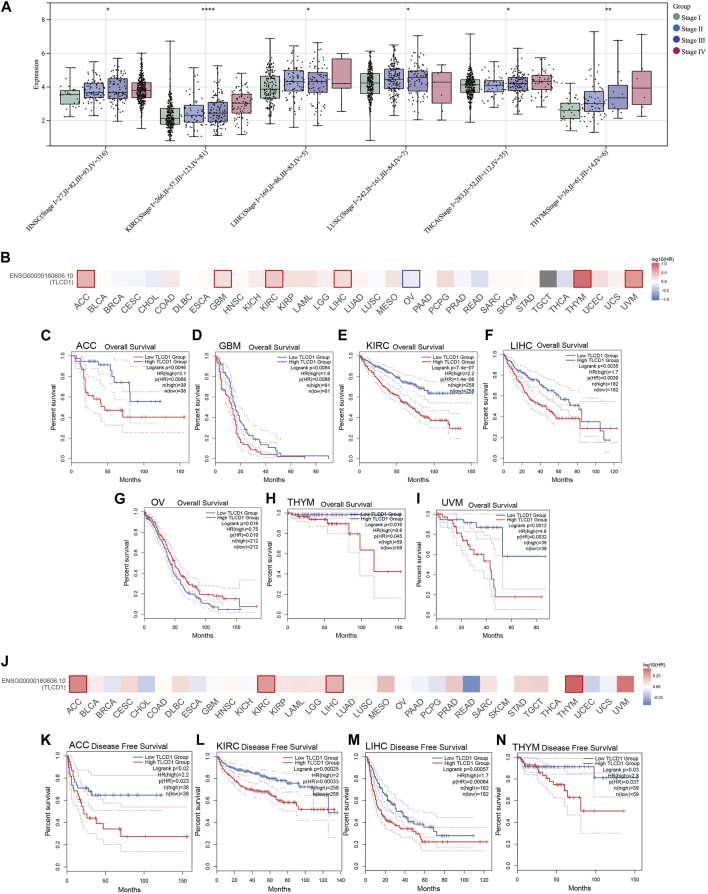
Clinical characterization of TLCD1 in pan-cancer. **(A)** Correlation between TLCD1 expression and cancer stage. **(B–I)** OS **(J–N)** and disease-free survival (DFS) analysis of different tumors in TCGA based on TLCD1 expression using the GEPIA2 tool. Red labels indicate positive correlation and blue labels indicate negative correlation. *p < 0.05, **p < 0.01, ***p < 0.001, ****p < 0.0001.

Our investigation aimed to determine whether TLCD1 expression was associated with tumor stage in a pan-cancer context. The analysis revealed a significant association between TLCD1 expression and tumor stage in several cancers, including HNSC, KIRC, LUSC, THYM, LIHC, THCA, and TGCT (Figure 4A). In addition, we evaluated the prognostic value of TLCD1 expression levels in various cancers by comparing high and low TLCD1 expression cohorts in terms of overall survival (OS) and disease-free survival (DFS). Using the GEPIA2 database and the TCGA pan-cancer cohort, our analysis showed that high TLCD1 mRNA expression levels were significantly associated with decreased OS in patients with ACC (HR = 3.1), GBM (HR = 2.2), KIRC (HR = 2.2), LIHC (HR = 1.7), OV(HR = 0.75), THYM (HR = 8.6) and UVM (HR = 4.6), however, in OV (HR = 0.75) patients, low TLCD1 mRNA expression levels were significantly associated with OS decline (Figures 4B–I). Similarly, DFS was significantly reduced in ACC (HR = 2.2), KIRC (HR = 2), LIHC (HR = 1.7) and THYM (HR = 2.8) patients with high TLCD1 mRNA expression levels (Figures 4J–N). These results suggest that elevated TLCD1 mRNA expression is associated with poorer prognosis in these cancers.

### 3.4 TLCD1 is tightly related to immune infiltration and immune checkpoints

**FIGURE 5:**
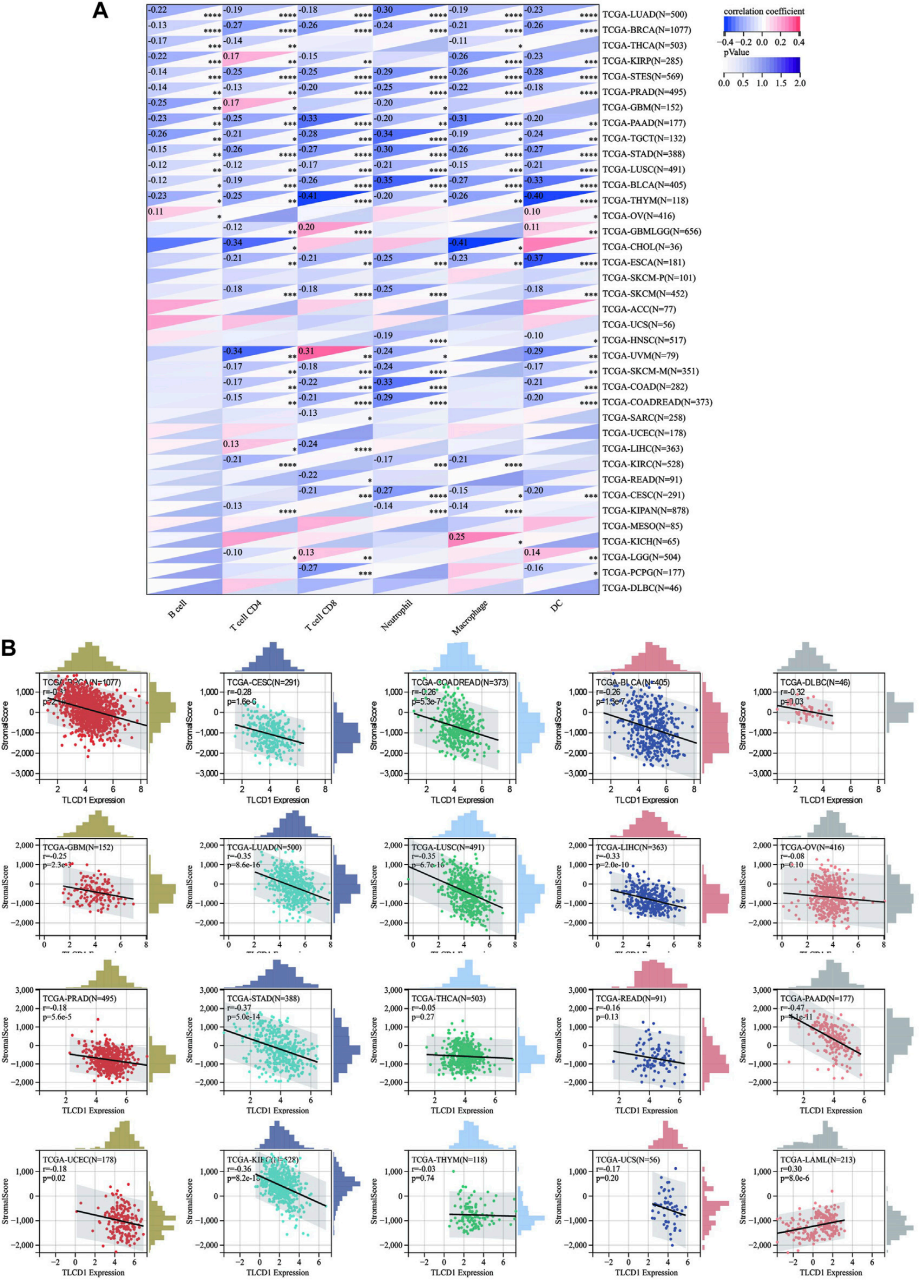
Correlation of TLCD1 expression with immune infiltration. **(A)** Relationship between TLCD1 expression and the degree of immune cell infiltration in a variety of malignant tumors using infiltration scores for six immune cell types (B cells, CD4cells, CD8cells, neutrophils, macrophages, and dendritic cells). *p < 0.05, **p < 0.01, ***p < 0.001, ****p < 0.0001. **(B)** Correlation between TLCD1 expression and StromalScore in various cancers.

**FIGURE 6:**
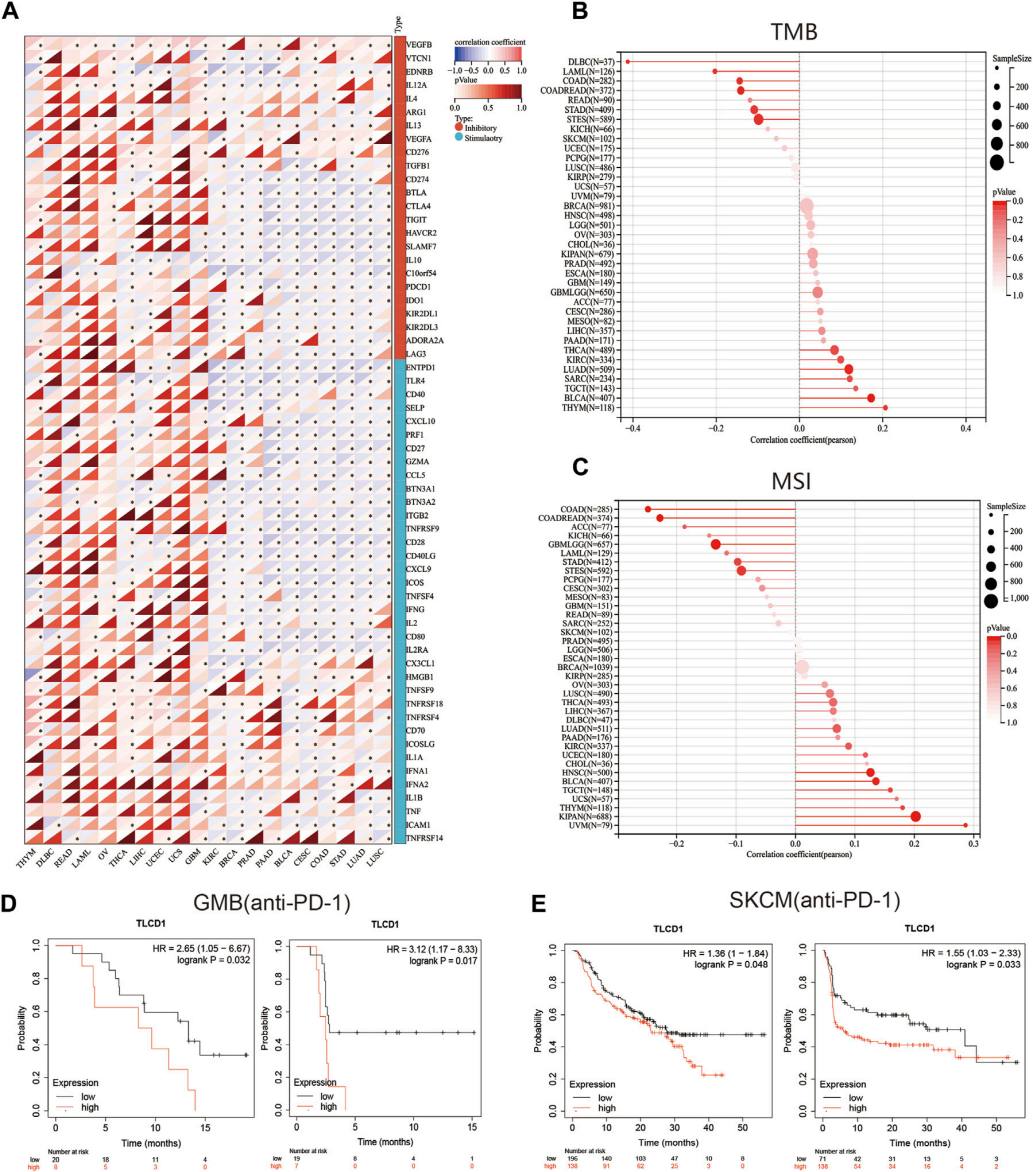
The correlation between TLCD1 expression and immune checkpoints. **(A)** Spearman correlation heatmap showing the correlation between TLCD1 and the expression of 60 genes of two types of immune checkpoint pathways (Inhibitory and Stimulatory) in pan-cancer. Red color represents positive correlation and blue color represents negative correlation. **(B)** Correlation between TLCD1 expression and tumor mutational load in pan-cancer. **(C)** Correlation between TLCD1 expression and microsatellite instability in pan-cancer. **(D)** Kaplan-Meier curves of low and high TLCD1 expression in GBM cancers treated with anti-PD-L1. **(E)** Kaplan-Meier curves of anti-PD-1 treated low and high TLCD1 melanoma patient groups. Marked asterisks indicate statistical *p* values (*p < 0.05, **p < 0.01, ***p < 0.001).

Our investigation delved into the significance of TLCD1 within the tumor microenvironment (TME) by exploring its association with pan-cancer immune infiltration levels. We used the immune infiltration timer module database of the Sanger tool to demonstrate the association of TLCD1 with various immune cell infiltrations (Figure 5A) and we examined the correlation between TLCD1 expression and the immune score known as StromalScore in various cancers. We analyzed cancer types where TLCD1 was significantly up or downregulated in tumor tissue (Figure 5B). According to the StromalScore analysis, TLCD1 expression showed a significant negative correlation with immune infiltration in BLCA, BRCA, CESC, COAD, DLBC, GBM, LIHC, LUAD, LUSC, OV, PAAD, PRAD, READ, STAD, THCA, THYM, UCEC, UCS, and KIRC. However, in LAML, immune infiltration levels increased with TLCD1 expression, suggesting that TLCD1 plays an important role in regulating the tumor immune microenvironment (TIME) in these malignancies. We further investigated the association between TLCD1 expression and 60 immune checkpoint pathway genes, divided into two classes (inhibitory and stimulatory) (Thorsson et al., 2018), using Spearman correlation analysis across the pan-cancer data (Figure 6A). Our analysis revealed that TLCD1 showed positive associations with most immunoregulatory genes in THYM, DLBC, READ, LAML, OV, THCA, LIHC, UCEC, UCS, and GBM. Conversely, it showed negative associations in KIRC, BRCA, PRAD, PAAD, BLCA, CESC, COAD, STAD, LUAD, and LUSC. To assess the potential of TLCD1 in predicting the efficacy of immune checkpoint inhibitor (ICI) therapy, we evaluated its correlation with two well-established predictive biomarkers for immunotherapy (Chan et al., 2019; Zhao et al., 2019), namely, tumor mutational burden (TMB) and microsatellite instability (MSI) (Figures 6B, C). Our results showed that TLCD1 expression was positively correlated with TMB levels in THYM, BLCA, LUAD, SARC, TGCT and KIRC. Conversely, it showed negative correlations in DLBC, LAML, and COAD. TLCD1 expression was also positively correlated with MSI in UVM, KIPAN, THYM, and UCS, but negatively correlated in COAD, ACC, GBM, and LAML (Figure 6C). To evaluate the predictive role of TLCD1 in cancer immunotherapy, we examined its association with response to anti-PD1 therapy in GBM and SKCM patients. High TLCD1 expression was associated with worse survival and outcome compared to low TLCD1 expression, supporting the potential of TLCD1 as a biomarker for predicting immunotherapy response (Figures 6D, E). However, further comprehensive studies are needed to investigate the predictive role of TLCD1 in cancer immunotherapy for individual cancer types in a clinical and mechanistic context.

### 3.5 Drug sensitivity analysis of TLCD1

**FIGURE 7:**
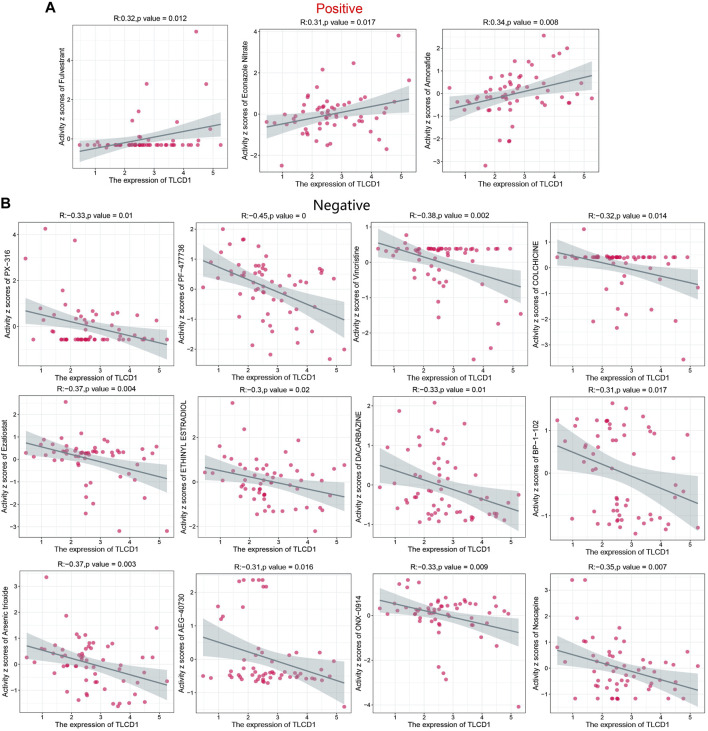
Drug sensitivity analysis of TLCD1. **(A,B)** Expression of TLCD1 and sensitivity to Vincristine,PX-316,PF-47736,Ezatiostat, ETHINYL ESTRADIOL, DACARBAZINE, Arsenic trioxide, AEG-40730, ONX-0914, COLCHICINE, BP-1-102, Noscapine, Fulvestrant, Econazole Nitrate and Amonafide.

Increased drug sensitivity is critical to prevent cancer cells from developing resistance to treatment. To explore this further, we performed a correlation analysis between drug sensitivity and TLCD1 expression levels using data from the CellMiner database. Our analysis revealed significant positive correlations between TLCD1 and drug sensitivity to fulvestrant, econazole nitrate, and amonafide, all of which are clinical drugs used in cancer treatment (Figure 7A). Conversely, TLCD1 showed significant but negative correlations with sensitivity to vincristine, PX-316, PF-47736, ezatiostat, ethinylestradiol, dacarbazine, arsenic trioxide, AEG-40730, ONX-0914, colchicine, BP-1-102, and noscapine (Figure 7B). These findings suggest that TLCD1 may be associated with chemoresistance to certain chemotherapeutic agents, including FDA-approved drugs such as fulvestrant and econazole nitrate. These results underscore the intricate relationship of TLCD1 with drug sensitivity in various cancer cell lines and position it as a promising therapeutic target in the field of cancer immunotherapy.

### 3.6 Analysis of TLCD1-related genes

**FIGURE 8:**
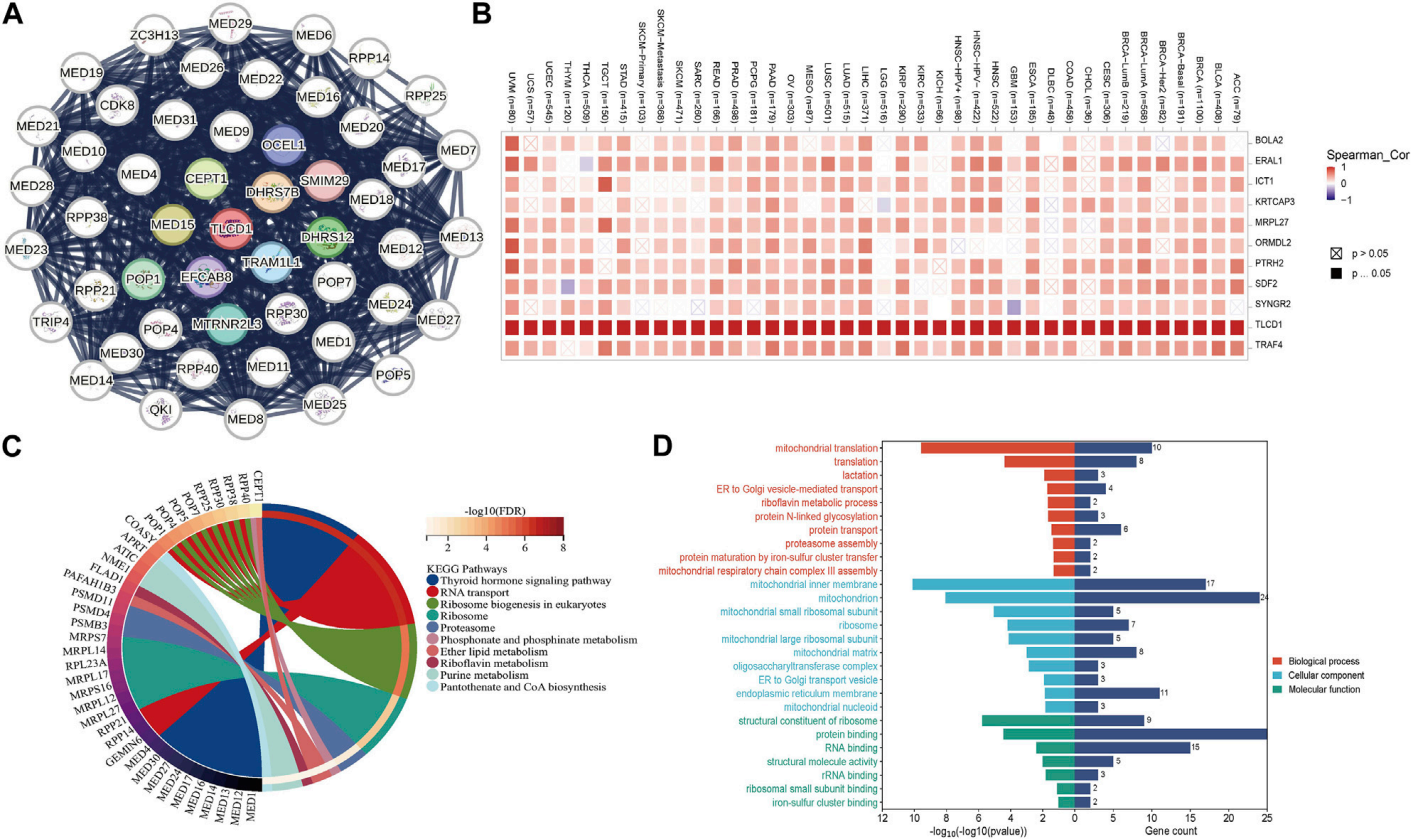
TLCD1-related gene enrichment analysis. **(A)** TLCD1-interacted proteins. **(B)** The expression of the top 10 TLCD1-related target genes in cancer. **(C)** KEGG pathway analysis based on the TLCD1-interacted and correlated genes. **(D)** GO analysis based on the TLCD1-interacted and correlated genes.

To gain deeper insights into the role of TLCD1 in tumorigenesis, we investigated the genes and proteins associated with TLCD1 expression and performed pathway enrichment analysis. First, we identified 50 TLCD1-binding proteins using the STRING online tool (Figure 8A) (Supplementary Table S1). In addition, we identified the top 100 TLCD1-associated target genes using GEPIA2 (Supplementary Table S2). The top 10 TLCD1-associated target genes were found to be positively correlated with tissue expression in different cancer types (Figure 8B), and we then performed KEGG and GO enrichment analyses using the identified genes. The KEGG results (Figure 8C) suggested that TLCD1 may exert its influence through pathways such as “thyroid hormone signaling pathway,” “RNA transport,” “ribosome biogenesis in eukaryotes,” “ribosome,” “phosphonate and phosphinate metabolism,” and “ether lipid metabolism,” all of which play a role in tumorigenesis. In terms of GO molecular functional analysis, most of the TLCD1-related genes were found to be related to processes involving “mitochondrial translation,” “mitochondrial inner membrane,” “mitochondrion,” and “structural component of ribosome” (Figure 8D). These findings shed light on the potential mechanisms by which TLCD1 influences tumorigenesis and its role in various cellular processes.

### 3.7 TLCD1 mutation characteristics in TCGA pan-cancer cohort

**FIGURE 9:**
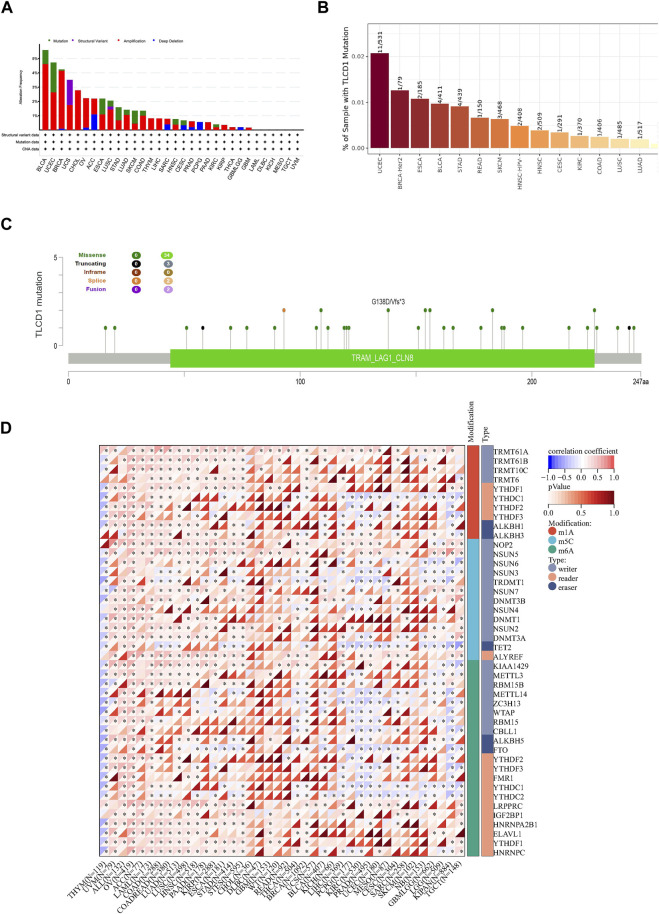
Genomic characterization of different cancers associated with TLCD1 expression. **(A)** Characterization of gene alterations (mutations, structural variants, amplifications, and deep deletions) of TLCD1 in 32 different tumors from the TCGA database was analyzed using the cBioPortal tool. Red labels represent the top 3 tumors in terms of amplification level. Green labels represent tumors with the highest mutation levels and blue labels represent tumors with the highest deep deletion levels; **(B)** mutation rate of TLCD1 gene in different tumors analyzed by TIMER portal; **(C)** mutation sites of TLCD1 gene in multiple tumors analyzed by cBioPortal tool. **(D)** Expression of the TLCD1 gene and 44 class III RNA modifier genes [m1A (Castro et al., 2016), m5C (Joyce and Pollard, 2009), m6A (Chan et al., 2019)] marker genes in each sample.

Understanding tumorigenesis requires a deeper exploration of genetic alterations, including mutations, structural variants, amplifications, and deep deletions affecting key genes (Martincorena et al., 2017). Therefore, we initiated an analysis of these diverse TLCD1 gene alterations using the TCGA cancer dataset accessible through cBioPortal. The most common TLCD1 gene alterations were amplifications in bladder urothelial carcinoma, uterine corpus endometrial carcinoma, breast cancer and uterine carcinosarcoma, and mutations in esophageal adenocarcinoma, lung squamous cell carcinoma, gastric carcinoma, lung adenocarcinoma, and cutaneous melanoma (Figure 9A). Analysis of the TIMER database revealed that ESCA (2/185), BRCA-Her2 (1/79) and UCEC (11/531) were the top three cancers with the highest rates of TLCD1 gene mutations (Figure 9B). Analysis of the cBioPortal database analysis showed that missense mutations were the main type of TLCD1 gene mutations in cancer (Figure 9C).

RNA modification plays an important role in cancer research because it regulates gene expression levels through several mechanisms, including N1-methyladenine (m1A), N6-methyladenine (m6A), and 5-methylcytosine (m5C). These modifications affect mRNA stability, translation, and expression of genes associated with cancer. Some RNA modifications have the potential to serve as cancer biomarkers, exhibiting distinct patterns in different cancer types and offering diagnostic and prognostic value. In addition, targeting rRNA modifications holds promise for cancer therapy, with m6A modification inhibitors potentially being used as a treatment strategy. Several studies have shown that regulating RNA modifications can improve the efficacy of cancer immunotherapy. To gain a deeper insight into the molecular mechanisms underlying the effect of the TLCD1 gene on cancer, we performed an analysis of TLCD1 gene expression together with 44 marker genes related to class III RNA modifications (m1A, m5C, m6A) in each sample Figure 9D. This comprehensive study of TLCD1 and RNA modifications contributes to a better understanding of the molecular mechanisms of cancer and provides new insights and avenues for cancer treatment.

## 4 Discussion

Mitochondria and cytoplasmic membranes are rich in phosphatidylethanolamine (PE) (van Meervan Meer and de Kroon, 2011). Research has shown that the composition of PE has significant implications for cellular metabolism (Richter-Dennerlein et al., 2014), oxidative stress (Doll et al., 2017; Kagan et al., 2017), and inflammatory signaling (Wenzel et al., 2017; Nagasaki et al., 2022). In addition, inhibiting the rate-limiting enzyme in the phosphatidylethanolamine biosynthetic pathway and combining it with the glycolysis inhibitor PFK158 to reprogram cancer cell metabolism can inhibit the growth of human liver cancer cells *in vitro* and *in vivo* (Guan et al., 2020). It is suggested that targeting the PE biosynthetic process may have some relevance to the study of cancer. Importantly, previous research has identified TLCD1 as a critical regulator of cellular PE composition. TLCD1 plays a role in facilitating the attachment of polyunsaturated fatty acids to the sn-1 position of PE. In addition, animal models have provided further validation, demonstrating that TLCD1/2 knockout leads to the development of fatty liver disease and a reduction in the incidence of non-alcoholic steatohepatitis (NASH) (Petkevicius et al., 2022). Therefore, this article focuses on TLCD1 to explore its relationship with pan-cancer, which has clinical value and significant guiding implications.

Pan-cancer analysis is of paramount importance in understanding the similarities and differences between different tumor types. It serves as a valuable approach to gain new insights into cancer prevention and targeted therapies that transcend specific cancer types. In recent years, the importance of comprehensive pan-cancer analyses has been increasingly recognized. Such analyses have the potential to reveal the critical role of specific driver mutations or genes in the initiation and progression of specific cancer types (Hong et al., 2020; Zhu et al., 2020). In this study, we demonstrated the tissue-specific expression of TLCD1 using data from the GTEx and HPA databases, consistent with previous findings. We then evaluated TLCD1 expression levels in different cancer types using multiple databases. The results showed increased TLCD1 mRNA expression in most cancer types compared to normal samples, including BLCA, BRCA, CESC, COAD, DLBC, GBM, LIHC, LUAD, LUSC, OV, PAAD, PRAD, READ, STAD, THCA, THYM, UCEC, and UCS, while decreased expression was observed in KIRC and LAML. It is possible that the low expression of TLCD1 in KIRC and LAML is caused by a variety of complex factors, including the biological characteristics of specific tumor types, gene regulatory mechanisms, genomic variations, and the influence of the tumor microenvironment. These findings strongly suggest that TLCD1 may indeed play a promoting role in cancer initiation and progression. Notably, the association between TLCD1 expression and cancer stage was examined and showed a significant correlation in cancers such as HNSC, KIRC, LUSC, THYM, LIHC, THCA, and TGCT. This suggests that TLCD1 expression levels may be a promising indicator to assess the stage of these cancers. In addition, we investigated whether TLCD1 could serve as a critical factor in cancer diagnosis and prognosis. Our analysis of TLCD1 expression levels in relation to overall survival (OS) and disease-free survival (DFS) in cancer patients revealed an association between increased TLCD1 expression in tumor tissues and poor prognosis. High TLCD1 expression was associated with adverse outcomes in cancers such as ACC, GBM, KIRC, LIHC, THYM and UVM. Thus, TLCD1 may prove valuable as a diagnostic and prognostic biomarker for personalized and precision cancer therapy.

Recent literature has highlighted the intricate relationship between the immune status of tumors, the components of the tumor tissue mesenchyme (TME), and the abundance of tumor-infiltrating immune cells (Yoshihara et al., 2013; Shiao et al., 2016). Using the ESTIMATE algorithm, we found that TLCD1 showed significant associations with immune infiltration and immunomodulatory genes. This suggests that TLCD1 may affect tumor development by influencing immune responses and immune infiltration within tumors. Recent studies have identified tumor mutational burden (TMB) and microsatellite instability (MSI) as predictive biomarkers for identifying patients who may benefit from immune checkpoint blockade therapy in various cancer types (Riaz et al., 2017; Rizvi et al., 2015; Le et al., 2017). Elevated TMB indicates genomic instability and is associated with improved response to tumor immunotherapy (Sabari et al., 2018; Li et al., 2020). Our study revealed correlations between aberrant TLCD1 expression and TMB in 12 cancer types and with MSI in 10 cancer types. These associations underscore the close relationship of TLCD1 with the tumor microenvironment and its potential as a biomarker for immunotherapy in specific cancer types. In addition, TLCD1 may be a target for cancer therapy. Unlike traditional chemotherapy, immune checkpoint inhibitors help restore the anti-tumor immune response. Analysis of the KMP database showed that high TLCD1 expression was associated with poorer survival and outcome in GBM and SKCM patients treated with anti-PD1 therapy. These findings highlight the potential of TLCD1 as a predictive biomarker for immunotherapy response.

We further explored the relationship between TLCD1 and drug sensitivity using the CellMiner database. This resource provides genomic and pharmacological tools for identifying drug response patterns and gene expressions across the NCI-60 cell line panel. Our investigation revealed significant positive correlations between TLCD1 expression and sensitivity to clinical anticancer drugs such as fulvestrant, econazole nitrate, and amofetil. Conversely, significant negative correlations were observed between TLCD1 expression and sensitivity to vincristine, PX-316, PF-47736, ezatiostat, ethinylestradiol, dacarbazine, arsenic trioxide, AEG-40730, ONX-0914, colchicine, BP-1-102, and noscapine. These results suggest a strong association between TLCD1 expression and sensitivity to anticancer treatment, indicating its potential as a therapeutic target. Our analysis also provided valuable insights into the function and mechanism of TLCD1 by exploring the enrichment of TLCD1-related genes in various functional and biological processes and by investigating potential mutation sites and types in TLCD1 itself. These findings contribute to a deeper understanding of the role of TLCD1 in cancer biology.

While our study sheds light on the role of TLCD1 in pan-cancer from a bioinformatics perspective, certain limitations should be acknowledged. First, although we have established a relationship between TLCD1 aberrant expression and immune cell infiltration and prognosis in human cancers, further investigation is required to elucidate the functional impact of TLCD1 on patient survival through immune responses. Therefore, the involvement of TLCD1 in immune regulation remains uncertain and warrants further research. Second, there is a lack of clinical trials evaluating the use of TLCD1-related therapeutic agents in pan-cancer patients. The drugs identified in our screening process require further experimental validation of their associations with various cancers and TLCD1. Therefore, prospective studies of TLCD1 expression and its role in cancer immune infiltration are critical to the development of new drugs.

## Figures and Tables

**Table:** 

**Cohort**	Full name
**TCGA-ACC**	Adrenocortical carcinoma
**TCGA-BLCA**	Bladder Urothelial Carcinoma
**TCGA-BRCA**	Breast invasive carcinoma
**TCGA-CESC**	Cervical squamous cell carcinoma and endocervical adenocarcinoma
**TCGA-CHOL**	Cholangiocarcinoma
**TCGA-COAD**	Colon adenocarcinoma
**TCGA-COADREAD**	Colon adenocarcinoma/Rectum adenocarcinoma Esophageal carcinoma
**TCGA-DLBC**	Lymphoid Neoplasm Diffuse Large B cell Lymphoma
**TCGA-ESCA**	Esophageal carcinoma
**TCGA-FPPP**	FFPE Pilot Phase II
**TCGA-GBM**	Glioblastoma multiforme
**TCGA-GBMLGG**	Glioma
**TCGA-HNSC**	Head and Neck squamous cell carcinoma
**TCGA-KICH**	Kidney Chromophobe
**TCGA-KIPAN**	Pan-kidney cohort (KICH + KIRC + KIRP)
**TCGA-KIRC**	Kidney renal clear cell carcinoma
**TCGA-KIRP**	Kidney renal papillary cell carcinoma
**TCGA-LAML**	Acute Myeloid Leukemia
**TCGA-LGG**	Brain Lower Grade Glioma
**TCGA-LIHC**	Liver hepatocellular carcinoma
**TCGA-LUAD**	Lung adenocarcinoma
**TCGA-LUSC**	Lung squamous cell carcinoma
**TCGA-MESO**	Mesothelioma
**TCGA-OV**	Ovarian serous cystadenocarcinoma
**TCGA-PAAD**	Pancreatic adenocarcinoma
**TCGA-PCPG**	Pheochromocytoma and Paraganglioma
**TCGA-PRAD**	Prostate adenocarcinoma
**TCGA-READ**	Rectum adenocarcinoma
**TCGA-SARC**	Sarcoma
**TCGA-STAD**	Stomach adenocarcinoma
**TCGA-SKCM**	Skin Cutaneous Melanoma
**TCGA-STES**	Stomach and Esophageal carcinoma
**TCGA-TGCT**	Testicular Germ Cell Tumors
**TCGA-THCA**	Thyroid carcinoma
**TCGA-THYM**	Thymoma
**TCGA-UCEC**	Uterine Corpus Endometrial Carcinoma
**TCGA-UCS**	Uterine Carcinosarcoma
**TCGA-UVM**	Uveal Melanoma

## Data Availability

The datasets presented in this study can be found in online repositories. The names of the repository/repositories and accession number(s) can be found in the article/Supplementary Material.
